# Supplementary material for: Prevention of pancreatic acinar cell carcinoma by Roux-en-Y Gastric Bypass Surgery
Source: Nat Commun. 2018 Oct 10;9:4183. doi: 10.1038/s41467-018-06571-w (PMC6180124; doi:10.1038/s41467-018-06571-w)

Prevention of Pancreatic Acinar Cell Carcinoma by Roux-en-Y Gastric Bypass Surgery

Rui He[#1,2], Yue Yin[#1], Wenzhen Yin[1], Yin Li[1], Jing Zhao[1], Weizhen Zhang*[1,3]

Supplementary Table 1

List of antibody.

Antibody	Source	Catalogue number	Dilution
Tsc1	Cell Signaling Technology (Beverly, MA)	#4906	1:1000
phospho-mTOR	Cell Signaling Technology (Beverly, MA)	#2971	1:1000
mTOR	Cell Signaling Technology (Beverly, MA)	#2983	1:1000
phospho-S6	Cell Signaling Technology (Beverly, MA)	#4858	1:1000
S6	Cell Signaling Technology (Beverly, MA)	#2217	1:1000
p21	Cell Signaling Technology (Beverly, MA)	#2947	1:1000
p27	Cell Signaling Technology (Beverly, MA)	#3686	1:1000
p53	Cell Signaling Technology (Beverly, MA)	#2524	1:1000
β-actin	Santa Cruz Biotechnology Inc. (Santa Cruz, CA)	sc-47778	1:2000

Supplementary Figure 1

Plasma levels of Glucagon-like peptide 1 (GLP-1)

Plasma was harvested after surgery at the age of 44 weeks and levels of GLP-1 measured according to the manufacturer's instruction. Values are mean±SEM. n=9,5,5 for WT sham, *Ngn3-Tsc1*-/-(KO) sham, *Ngn3-Tsc1*-/-(KO) RYGB respectively. Statistical differences were analyzed by one-way ANOVA followed by the Student's t-test. #P<0.05 *Ngn3-Tsc1*-/-(KO) sham vs WT sham, *P<0.05 *Ngn3-Tsc1*-/-(KO) RYGB vs *Ngn3-Tsc1*-/-(KO) sham.

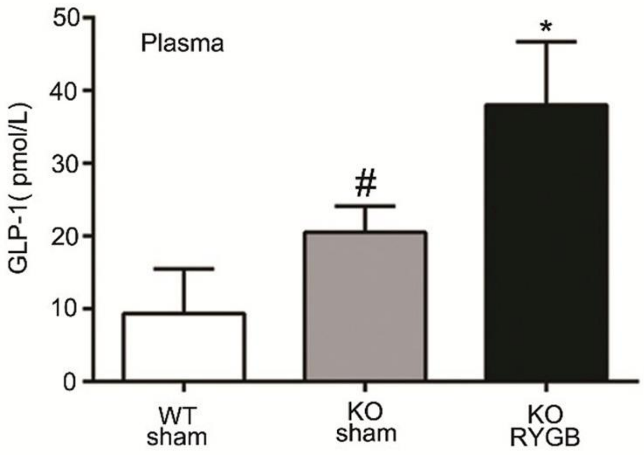

Supplementary Figure 2

Uncropped scans of the most important blots:

1. Tsc1

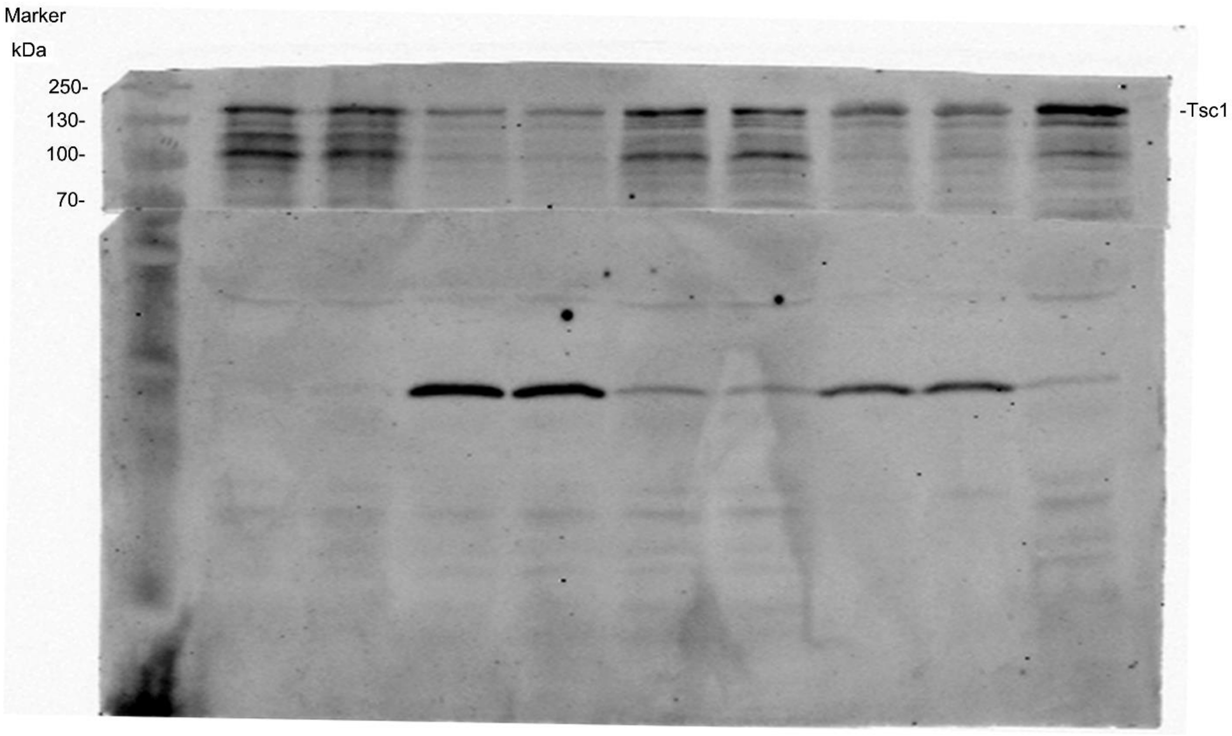

2. mTOR

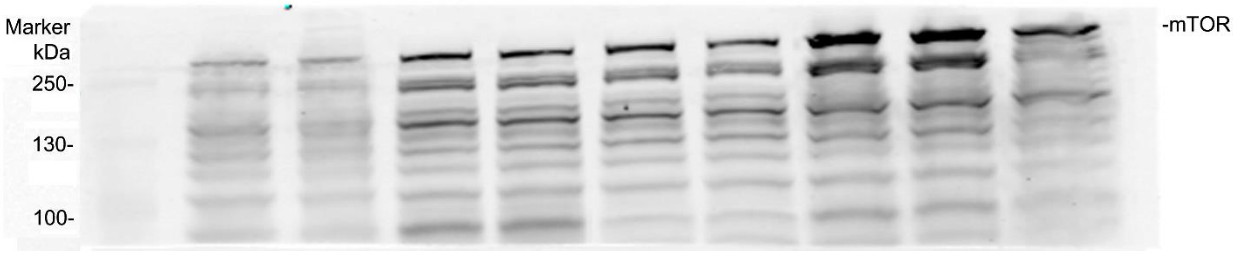

3. p-mTOR

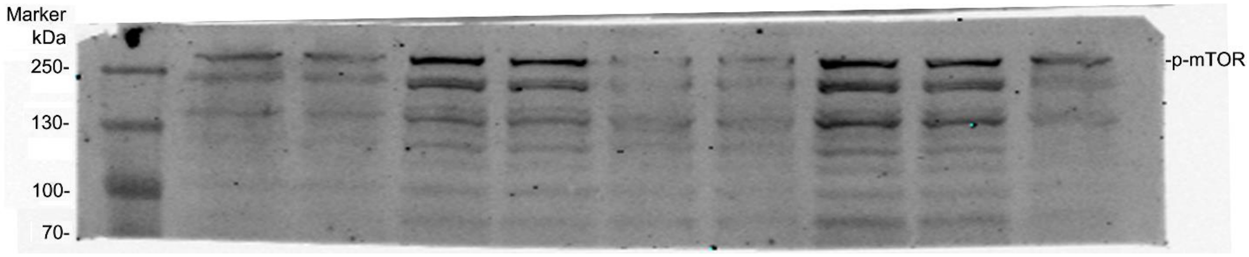

4. p-S6

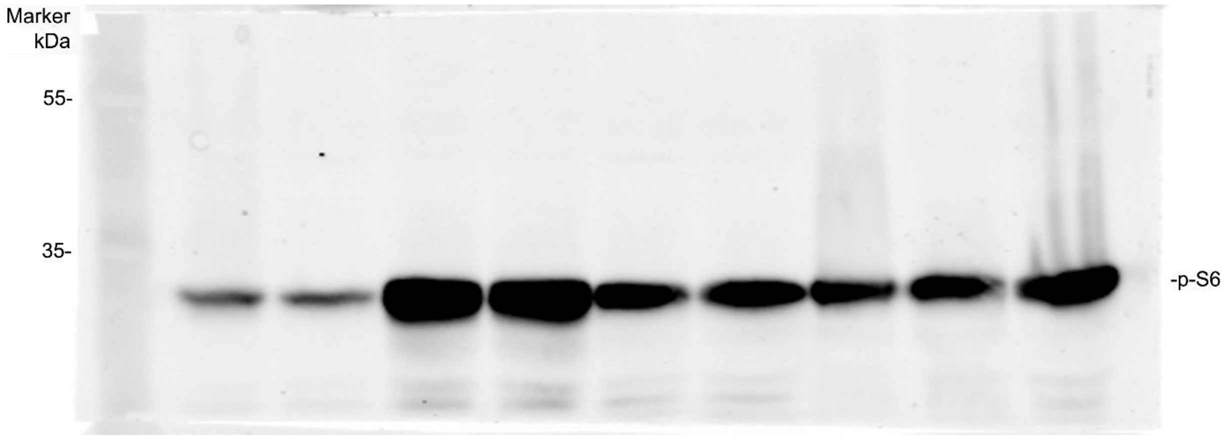

5. S6

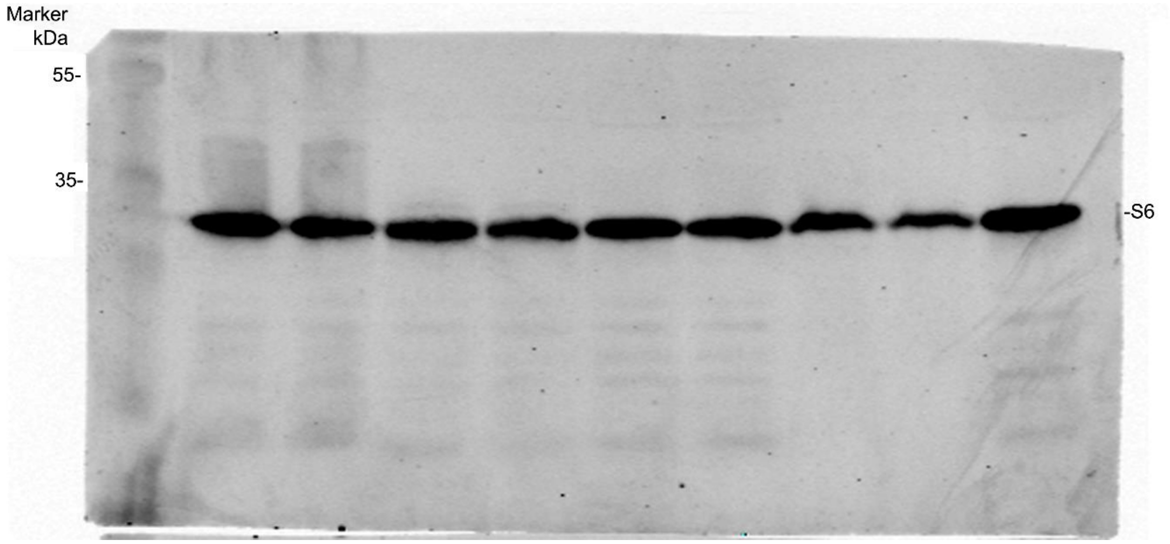

6. p21

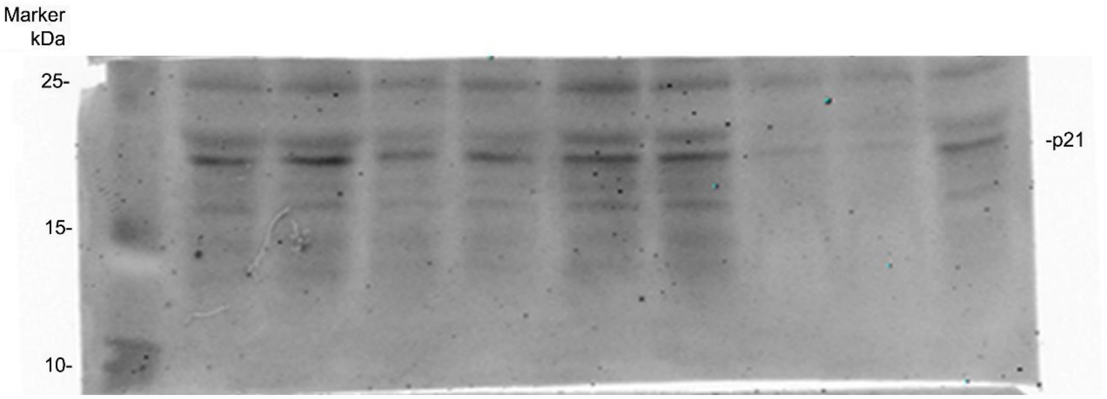

7. p27

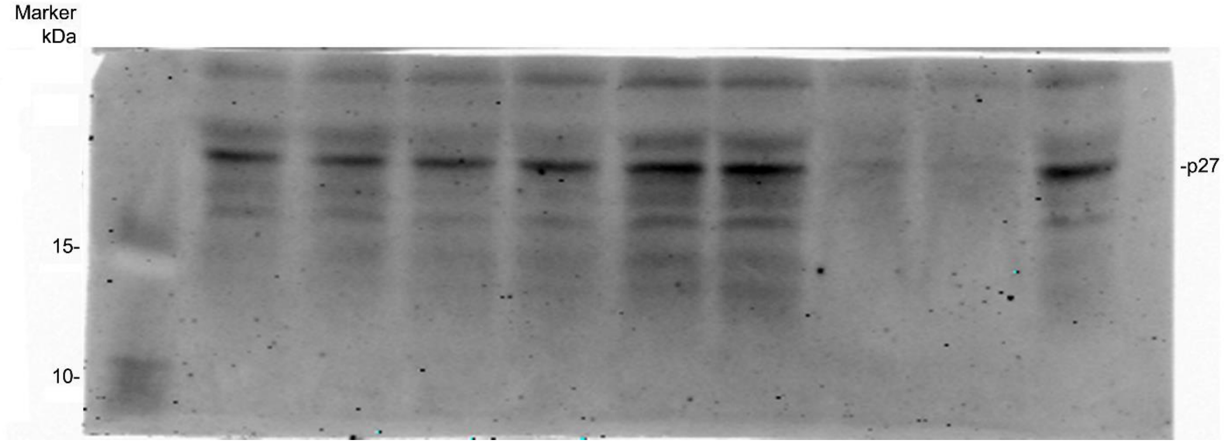

8. p53

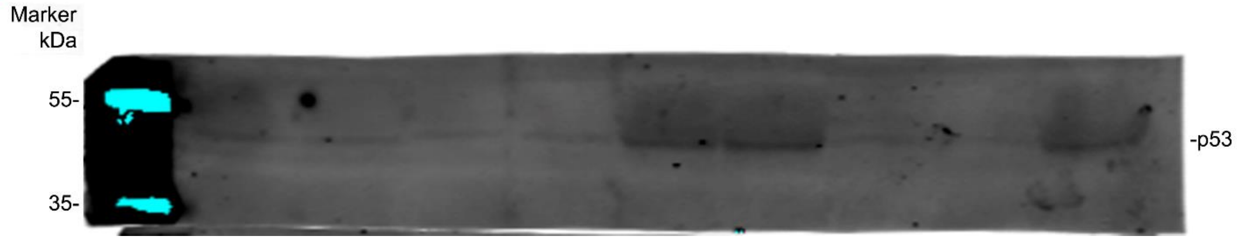

9. β-actin

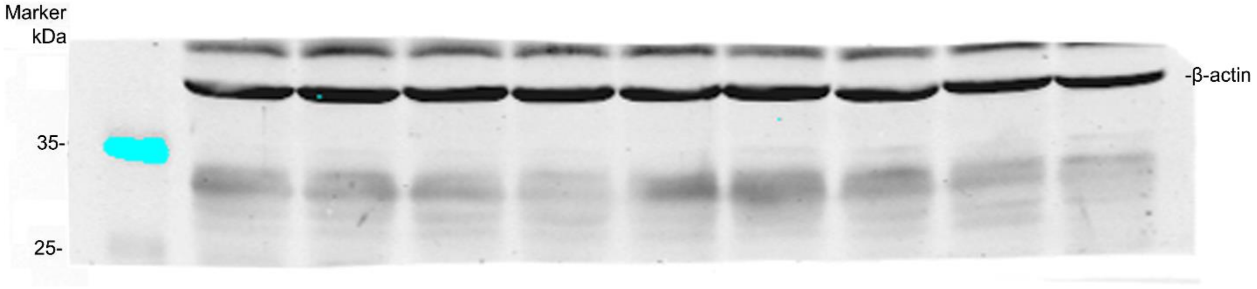

Supplement: Supplementary file 1 — Supplementary Information [file 41467_2018_6571_MOESM1_ESM.pdf]
